# Supplementary material for: Global report on COVID-19 vaccination and reasons not to vaccinate among adults with intellectual disabilities: Results from secondary analyses of Special Olympics’ program planning
Source: PLOS Glob Public Health. 2023 Jun 13;3(6):e0001367. doi: 10.1371/journal.pgph.0001367 (PMC10263337; doi:10.1371/journal.pgph.0001367)
Supplement: S2 Table — (DOCX) [file pgph.0001367.s002.docx]

**Supplemental Table 2. Number of respondents by country grouped by country income level.**

| **Country Income Level** | **Frequency of Respondents** |
| --- | --- |
| **Low-Income** | |
| Afghanistan | 4 |
| Burkina Faso | 41 |
| Burundi | 43 |
| Congo, Dem. Rep | 5 |
| Ethiopia | 3 |
| Gambia | 16 |
| Guinea | 10 |
| Madagascar | 62 |
| Malawi | 8 |
| Mali | 2 |
| Mozambique | 5 |
| Niger | 1 |
| Rwanda | 73 |
| Somalia | 3 |
| Sudan | 54 |
| Togo | 2 |
| Uganda | 77 |
| Yemen | 1 |
| **Lower-Middle Income** | |
| Algeria | 58 |
| Bangladesh | 54 |
| Benin | 10 |
| Bolivia | 7 |
| Congo, Rep. | 5 |
| Djibouti | 1 |
| Egypt | 36 |
| El Salvador | 10 |
| Eswatini | 24 |
| Ghana | 35 |
| Honduras | 37 |
| India | 1 |
| Indonesia | 16 |
| Iran | 119 |
| Ivory Coast | 8 |
| Kenya | 170 |
| Kyrgyz Republic | 3 |
| Laos | 1 |
| Lesotho | 37 |
| Mauritania | 112 |
| Mongolia | 3 |
| Morocco | 29 |
| Nicaragua | 6 |
| Nigeria | 35 |
| Pakistan | 4 |
| Palestine | 1 |
| Papua New Guinea | 5 |
| Samoa | 6 |
| Senegal | 2 |
| Tanzania | 222 |
| Tunisia | 46 |
| Uzbekistan | 5 |
| Vietnam | 1 |
| Zambia | 23 |
| Zimbabwe | 50 |
| **Upper-Middle Income** | |
| Albania | 2 |
| American Samoa | 4 |
| Argentina | 42 |
| Armenia | 3 |
| Azerbaijan | 1 |
| Belarus | 1 |
| Bosnia and Herzegovina | 1 |
| Botswana | 16 |
| Brazil | 70 |
| Bulgaria | 2 |
| China | 57 |
| Costa Rica | 18 |
| Dominican Republic | 21 |
| Ecuador | 19 |
| Equatorial Guinea | 2 |
| Fiji | 2 |
| Georgia | 1 |
| Guatemala | 36 |
| Iraq | 28 |
| Jordan | 4 |
| Kazakhstan | 2 |
| Kosovo | 1 |
| Lebanon | 38 |
| Malaysia | 30 |
| Maldives | 1 |
| Mauritius | 37 |
| Mexico | 88 |
| Montenegro | 1 |
| Namibia | 4 |
| North Macedonia | 1 |
| Panama | 47 |
| Paraguay | 17 |
| Peru | 35 |
| Romania | 1 |
| Russia | 77 |
| Serbia | 6 |
| South Africa | 65 |
| Thailand | 3 |
| Turkey | 1 |
| Turkmenistan | 2 |
| Venezuela | 50 |
| **High-Income** | |
| Andorra | 7 |
| Australia | 67 |
| Austria | 19 |
| Bahamas | 1 |
| Bahrain | 50 |
| Belgium | 1 |
| Brunei Darussalam | 1 |
| Canada | 71 |
| Croatia | 3 |
| Cyprus | 13 |
| Czech Republic | 3 |
| Estonia | 4 |
| Faroe Islands | 2 |
| Finland | 17 |
| France | 2 |
| Germany | 22 |
| Gibraltar | 3 |
| Hong Kong | 5 |
| Isle of Man | 1 |
| Korea, Rep. | 1 |
| Kuwait | 34 |
| Liechtenstein | 1 |
| Lithuania | 2 |
| Luxembourg | 2 |
| Macau | 12 |
| Monaco | 1 |
| Netherlands | 8 |
| Oman | 9 |
| Poland | 1 |
| Portugal | 11 |
| Qatar | 36 |
| Saudi Arabia | 37 |
| Seychelles | 46 |
| Singapore | 1 |
| Slovakia | 1 |
| Slovenia | 1 |
| Spain | 53 |
| Sweden | 1 |
| Switzerland | 18 |
| Taiwan, China | 15 |
| United Arab Emirates | 11 |
| United Kingdom | 7 |
| United States | 526 |
| Uruguay | 4 |
