# Supplementary material for: Global report on COVID-19 vaccination and reasons not to vaccinate among adults with intellectual disabilities: Results from secondary analyses of Special Olympics’ program planning
Source: PLOS Glob Public Health. 2023 Jun 13;3(6):e0001367. doi: 10.1371/journal.pgph.0001367 (PMC10263337; doi:10.1371/journal.pgph.0001367)
Supplement: S3 Table — (DOCX) [file pgph.0001367.s003.docx]

**Supplemental Table 3.** Univariate and multivariate logistic regression models of vaccination, booster, and infection among Special Olympics global survey respondents (n=3560).

|  | **Logit Coefficient**  **[95% CI]** | **Wald z-statistic**  **(*p*-value)** | **OR**  **[95% CI]** |
| --- | --- | --- | --- |
| **Vaccination – Univariate** | | | |
| Age (year) | 0.076  [0.065, 0.088] | 12.6  (p < 0.001) | 1.08  [1.07, 1.09] |
| Country economic income level | 1.221  [1.120, 1.326] | 23.3  (*p* < 0.001) | 3.39  [3.07, 3.76] |
| Live with family | -0.642  [-0.883, -0.411] | -5.3  (p < 0.001) | 0.53  [0.41, 0.66] |
| Male | 0.138  [-0.019, 0.295] | 1.7  (*p* = 0.084) | 1.15  [0.98, 1.34] |
| Type of intellectual disability |  |  |  |
| Autism | -0.010  [-0.204, 0.189] | -0.1  (*p* = 0.924) | 0.99  [0.82, 1.21] |
| Down syndrome | -0.099  [-0.268, 0.071] | -1.1  (*p* = 0.251) | 0.91  [0.77, 1.07] |
| Fragile X | -0.423  [-0.768, -0.063] | -2.4  (p = 0.019) | 0.66  [0.46, 0.94] |
| Cerebral palsy | -1.015  [-1.285, -0.742] | -7.3  (p < 0.001) | 0.36  [0.28, 0.48] |
| Other | 0.474  [0.314, 0.636] | 5.8  (*p* < 0.001) | 1.61  [1.37, 1.89] |
| **Vaccination – Multivariate**  Age (year)  Country economic income level  Live with family  Male  Autism  Down syndrome  Fragile X  Cerebral palsy  Other | 0.038  [0.025, 0.051]  1.138  [1.032, 1.248]  -0.354  [-0.630, -0.085]  0.178  [-0.002, 0.358]  0.280  [-0.318, 0.942]  0.350  [-0.266, 1.029]  -0.277  [-1.004, 0.506]  -0.672  [-1.323, 0.036]  0.200  [-0.427, 0.891] | 5.6  (*p* < 0.001)  20.7  (*p* < 0.001)  -2.5  (*p* = 0.011)  1.9  (*p* = 0.052)  0.9  (*p* = 0.383)  1.1  (*p* = 0.289)  -0.7  (*p* = 0.472)  -1.9  (*p* = 0.052)  0.6  (*p* = 0.551) | 1.04  [1.03, 1.05]  3.12  [2.81, 3.48]  0.70  [0.53, 0.92]  1.20  [1.00, 1.43]  1.32  [0.73, 2.57]  1.42  [0.77, 2.80]  0.76  [0.37, 1.66]  0.51  [0.27, 1.04]  1.22  [0.65, 2.44] |
| **Boosted – Univariate** | | | |
| Age (year) | 0.075  [0.066, 0.084] | 16.5  (p < 0.001) | 1.08  [1.07, 1.09] |
| Country economic income level | 1.094  [1.013, 1.176] | 26.3  (*p* < 0.001) | 2.99  [2.75, 3.24] |
| Live with family | -0.369  [-0.546, -0.193] | -4.1  (p < 0.001) | 0.69  [0.58, 0.82] |
| Male | 0.059  [-0.075, 0.193] | 0.9  (*p* = 0.386) | 1.06  [0.93, 1.21] |
| Type of intellectual disability |  |  |  |
| Autism | -0.143  [-0.204, 0.189] | -1.7  (*p* = 0.095) | 0.87  [0.73, 1.03] |
| Down syndrome | 0.018  [-0.128, 0.164] | 0.2  (*p* = 0.811) | 1.02  [0.88, 1.18] |
| Fragile X | -0.656  [-1.005, -0.319] | -3.8  (p < 0.001) | 0.52  [0.37, 0.73] |
| Cerebral palsy | -0.657  [-0.939, -0.382] | -4.6  (p < 0.001) | 0.52  [0.39, 0.68] |
| Other | 0.326  [0.192, 0.459] | 4.8  (*p* < 0.001) | 1.39  [1.21, 1.58] |
| **Boosted – Multivariate**  Age (year)  Country economic income level  Live with family  Male  Autism  Down syndrome  Fragile X  Cerebral palsy  Other | 0.049  [0.039, 0.059]  1.014  [0.928, 1.100]  0.096  [-0.120, 0.312]  0.128  [-0.029, 0.285]  0.040  [-0.500, 0.571]  0.395  [-0.160, 0.942]  -0.904  [-1.564, -0.249]  -0.401  [-0.991, 0.182]  0.140  [-0.421, 0.692] | 9.5  (*p* < 0.001)  23.1  (*p* < 0.001)  0.9  (*p* = 0.383)  1.6  (*p* = 0.112)  0.1  (*p* = 0.882)  1.4  (*p* = 0.153)  -2.7  (*p* = 0.006)  -1.4  (*p* = 0.174)  0.5  (*p* = 0.616) | 1.05  [1.04, 1.06]  2.76  [2.53, 3.01]  1.10  [0.89, 1.37]  1.14  [0.97, 1.33]  1.04  [0.61, 1.77]  1.48  [0.85, 2.56]  0.40  [0.21, 0.78]  0.67  [0.37, 1.20]  1.15  [0.66, 2.00] |
| **Infection – Univariate** | | | |
| Age (year) | 0.017  [0.008, 0.027] | 3.5  (p < 0.001) | 1.02  [1.01, 1.03] |
| Country economic income level | 0.425  [0.332, 0.519] | 8.9  (p < 0.001) | 1.53  [1.39, 1.68] |
| Live with family | -0.375  [-0.594, -0.150] | -3.3  (p < 0.001) | 0.69  [0.55, 0.86] |
| Male | -0.009  [-0.191, 0.174] | -0.1  (*p* = 0.922) | 0.99  [0.83, 1.19] |
| Type of intellectual disability |  |  |  |
| Autism | 0.101  [-0.126, 0.322] | 0.9  (*p* = 0.375) | 1.11  [0.88, 1.38] |
| Down syndrome | -0.215  [-0.424, -0.012] | -2.1  (*p* = 0.040) | 0.81  [0.65, 0.99] |
| Fragile X | -0.179  [-0.675, 0.269] | -0.7  (*p* = 0.456) | 0.84  [0.51, 1.31] |
| Cerebral palsy | -0.352  [-0.778, 0.036] | -1.7  (*p* = 0.089) | 0.70  [0.46, 1.04] |
| Other | 0.185  [0.004, 0.365] | 2.0  (*p* = 0.044) | 1.20  [1.00, 1.44] |
| **Infection – Multivariate**  Age (year)  Country economic    income level  Live with family  Male  Autism  Down syndrome  Fragile X  Cerebral palsy  Other | 0.001  [-0.010, 0.012]  0.402  [0.302, 0.502]  -0.232  [-0.464, 0.006]  0.006  [-0.180, 0.193]  -0.296  [-1.242, 0.417]  -0.456  [-1.416, 0.274]  -0.550  [-1.591, 0.284]  -0.605  [-1.595, 0.168]  -0.375  [-1.339, 0.361] | 0.2  (*p* = 0.857)  7.9  (*p* < 0.001)  -1.9  (*p* = 0.053)  0.1  (*p* = 0.952)  -0.7  (*p* = 0.477)  -1.1  (*p* = 0.284)  -1.2  (*p* = 0.246)  -1.4  (*p* = 0.173)  -0.9  (*p* = 0.381) | 1.00  [0.99, 1.01]  1.49  [1.35, 1.65]  0.79  [0.63, 1.01]  1.01  [0.84, 1.21]  0.74  [0.29, 1.52]  0.63  [0.24, 1.32]  0.58  [0.20, 1.33]  0.55  [0.20, 1.18]  0.69  [0.26, 1.44] |
